# (2-Amido-3-oxidopyridinium-κ^2^ *N*,*O*)dibenzyl­chloridotin(IV)

**DOI:** 10.1107/S1600536809020339

**Published:** 2009-06-06

**Authors:** Chui Lian Tan, Kong Mun Lo, Seik Weng Ng

**Affiliations:** a Department of Chemistry, University of Malaya, 50603 Kuala Lumpur, Malaysia

## Abstract

The Sn atom in the title compound, [Sn(C_7_H_7_)_2_(C_5_H_5_N_2_O)Cl], shows a distorted C_2_ClNOSn trigonal-bipyramidal coordin­ation, with a Cl–Sn–O axial angle of 163.77 (3)°, but the C—Sn—C angle [141.43 (7)°] deviates from 120°. The chelating ligand exists in a zwitterionic form. Adjacent molecules are linked by an N—H_pyridinium_⋯O hydrogen bond, forming a chain running along the *c* axis of the orthorhombic unit cell.

## Related literature

2-Amino-3-hydroxy­pyridine behaves as a mono-anion chelating to a metal atom; see: Gerber *et al.* (2004 ▶). The ligand also chelates in the neutral form; see: Palkina *et al.* (2000 ▶). The ligand exists as an isolated mono-cation in other metal salts; see: Halvorson *et al.* (1990 ▶); Place *et al.* (1998 ▶).
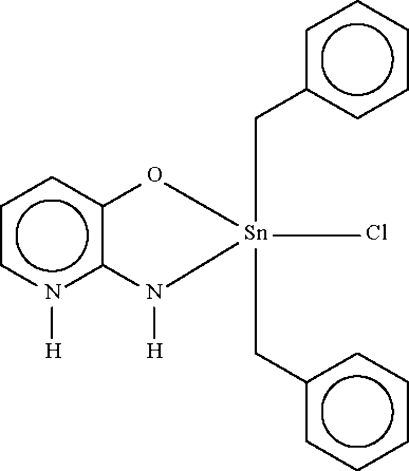


## Experimental

### 

#### Crystal data

[Sn(C_7_H_7_)_2_(C_5_H_5_N_2_O)Cl]
*M* *_r_* = 445.50
Orthorhombic, 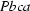

*a* = 11.0457 (1) Å
*b* = 16.8447 (2) Å
*c* = 19.1227 (2) Å
*V* = 3558.00 (6) Å^3^
*Z* = 8
Mo *K*α radiation
μ = 1.59 mm^−1^
*T* = 133 K
0.20 × 0.05 × 0.05 mm

#### Data collection

Bruker SMART APEX diffractometer
Absorption correction: multi-scan (*SADABS*; Sheldrick, 1996 ▶) *T*_min_ = 0.640, *T*_max_ = 0.746 (expected range = 0.792–0.923)
32398 measured reflections
4086 independent reflections
3434 reflections with *I* > 2σ(*I*)
*R*_int_ = 0.025

#### Refinement

*R*[*F* ^2^ > 2σ(*F* ^2^)] = 0.021
*wR*(*F* ^2^) = 0.054
*S* = 1.01
4086 reflections
225 parameters
2 restraints
H atoms treated by a mixture of independent and constrained refinement
Δρ_max_ = 0.58 e Å^−3^
Δρ_min_ = −0.38 e Å^−3^

### 

Data collection: *APEX2* (Bruker, 2007 ▶); cell refinement: *SAINT* (Bruker, 2007 ▶); data reduction: *SAINT*; program(s) used to solve structure: *SHELXS97* (Sheldrick, 2008 ▶); program(s) used to refine structure: *SHELXL97* (Sheldrick, 2008 ▶); molecular graphics: *X-SEED* (Barbour, 2001 ▶); software used to prepare material for publication: *publCIF* (Westrip, 2009 ▶).

## Supplementary Material

Crystal structure: contains datablocks global, I. DOI: 10.1107/S1600536809020339/tk2469sup1.cif

Structure factors: contains datablocks I. DOI: 10.1107/S1600536809020339/tk2469Isup2.hkl

Additional supplementary materials:  crystallographic information; 3D view; checkCIF report

## Figures and Tables

**Table 1:** Hydrogen-bond geometry (Å, °)

*D*—H⋯*A*	*D*—H	H⋯*A*	*D*⋯*A*	*D*—H⋯*A*
N1—H1⋯O1^i^	0.88 (1)	1.87 (1)	2.726 (2)	165 (2)

Symmetry code: (i) 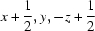.

**Fig. 1.**
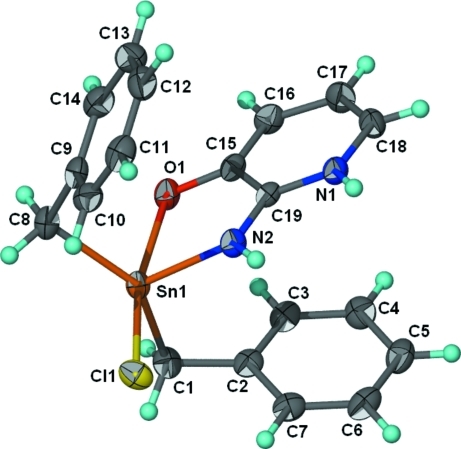
Thermal ellipsoid plot (Barbour, 2001) of SnCl(C7H7)(C5H5N2O) at the 70% probability level. Hydrogen atoms are drawn as spheres of arbitrary radius.

## supplementary crystallographic information

### Experimental

Dibenzyltin dichloride (0.37 g, 1 mmol) and 2-amino-3-hydroxypyridine (0.11 g, 1 mmol) were dissolved in chloroform (100 ml); the solution was heated for 1 hour. Slow evaporation of the filtrate afforded pale-yellow crystals.

### Refinement

Hydrogen atoms were placed at calculated positions (C–H 0.95–0.99 Å) and were treated as riding on their parent atoms, with *U*(H) set to 1.2*U*_eq_(C). The nitrogen-bound H atoms were located in a difference Fourier map, and were refined with a distance restraint of N–H 0.88±0.01 Å and individual isotropic temperature factors.

### Crystal data

**Table:**

[Sn(C_7_H_7_)_2_(C_5_H_5_N_2_O)Cl]	*F*(000) = 1776
*M**_r_* = 445.50	*D*_x_ = 1.663 Mg m^−^^3^
Orthorhombic, *P**b**c**a*	Mo *K*α radiation, λ = 0.71073 Å
Hall symbol: -P 2ac 2ab	Cell parameters from 9941 reflections
*a* = 11.0457 (1) Å	θ = 2.4–28.2°
*b* = 16.8447 (2) Å	µ = 1.59 mm^−^^1^
*c* = 19.1227 (2) Å	*T* = 133 K
*V* = 3558.00 (6) Å^3^	Prism, pale-yellow
*Z* = 8	0.20 × 0.05 × 0.05 mm

### Data collection

**Table:**

Bruker SMART APEX diffractometer	4086 independent reflections
Radiation source: fine-focus sealed tube	3434 reflections with *I* > 2σ(*I*)
graphite	*R*_int_ = 0.025
ω scans	θ_max_ = 27.5°, θ_min_ = 2.1°
Absorption correction: multi-scan (*SADABS*; Sheldrick, 1996)	*h* = −14→14
*T*_min_ = 0.640, *T*_max_ = 0.746	*k* = −21→21
32398 measured reflections	*l* = −24→24

### Refinement

**Table:**

Refinement on *F*^2^	Primary atom site location: structure-invariant direct methods
Least-squares matrix: full	Secondary atom site location: difference Fourier map
*R*[*F*^2^ > 2σ(*F*^2^)] = 0.021	Hydrogen site location: inferred from neighbouring sites
*wR*(*F*^2^) = 0.054	H atoms treated by a mixture of independent and constrained refinement
*S* = 1.01	*w* = 1/[σ^2^(*F*_o_^2^) + (0.0279*P*)^2^ + 2.1692*P*] where *P* = (*F*_o_^2^ + 2*F*_c_^2^)/3
4086 reflections	(Δ/σ)_max_ = 0.004
225 parameters	Δρ_max_ = 0.58 e Å^−^^3^
2 restraints	Δρ_min_ = −0.38 e Å^−^^3^

### Fractional atomic coordinates and isotropic or equivalent isotropic displacement parameters (Å^2^)

**Table:**

	*x*	*y*	*z*	*U*_iso_*/*U*_eq_	
Sn1	0.497920 (10)	0.547145 (7)	0.324875 (6)	0.01725 (5)	
Cl1	0.65272 (4)	0.45738 (3)	0.37607 (3)	0.02492 (10)	
O1	0.40585 (10)	0.64531 (8)	0.26870 (6)	0.0207 (3)	
N1	0.68659 (13)	0.70633 (9)	0.19394 (8)	0.0185 (3)	
H1	0.7626 (10)	0.6937 (12)	0.2015 (11)	0.024 (5)*	
N2	0.63839 (13)	0.61218 (9)	0.28027 (8)	0.0183 (3)	
H2	0.7151 (10)	0.6008 (13)	0.2832 (12)	0.033 (6)*	
C1	0.43387 (17)	0.46293 (11)	0.24812 (10)	0.0224 (4)	
H1A	0.4448	0.4079	0.2653	0.027*	
H1B	0.3469	0.4716	0.2383	0.027*	
C2	0.50788 (15)	0.47652 (12)	0.18346 (10)	0.0212 (4)	
C3	0.46973 (18)	0.53013 (12)	0.13232 (10)	0.0230 (4)	
H3	0.3931	0.5552	0.1369	0.028*	
C4	0.54191 (19)	0.54737 (12)	0.07487 (11)	0.0261 (4)	
H4	0.5147	0.5844	0.0408	0.031*	
C5	0.65348 (18)	0.51080 (12)	0.06696 (10)	0.0277 (4)	
H5	0.7032	0.5228	0.0278	0.033*	
C6	0.69168 (18)	0.45658 (12)	0.11679 (11)	0.0274 (4)	
H6	0.7676	0.4308	0.1114	0.033*	
C7	0.62000 (17)	0.43961 (12)	0.17456 (10)	0.0238 (4)	
H7	0.6476	0.4025	0.2084	0.029*	
C8	0.44732 (17)	0.60092 (12)	0.42300 (10)	0.0229 (4)	
H8A	0.3655	0.6246	0.4198	0.027*	
H8B	0.4474	0.5609	0.4609	0.027*	
C9	0.54035 (17)	0.66398 (11)	0.43697 (9)	0.0216 (4)	
C10	0.65205 (17)	0.64382 (11)	0.46612 (9)	0.0234 (4)	
H10	0.6665	0.5907	0.4805	0.028*	
C11	0.74193 (18)	0.70028 (12)	0.47429 (10)	0.0271 (4)	
H11	0.8177	0.6854	0.4938	0.033*	
C12	0.72242 (19)	0.77828 (12)	0.45420 (11)	0.0286 (4)	
H12	0.7841	0.8170	0.4602	0.034*	
C13	0.6121 (2)	0.79920 (12)	0.42533 (11)	0.0301 (4)	
H13	0.5978	0.8526	0.4116	0.036*	
C14	0.52218 (18)	0.74232 (12)	0.41639 (11)	0.0265 (4)	
H14	0.4472	0.7572	0.3959	0.032*	
C15	0.48011 (15)	0.68662 (11)	0.22755 (10)	0.0179 (4)	
C16	0.44615 (17)	0.74253 (11)	0.17952 (10)	0.0225 (4)	
H16	0.3632	0.7565	0.1748	0.027*	
C17	0.53422 (18)	0.77968 (12)	0.13674 (10)	0.0246 (4)	
H17	0.5103	0.8175	0.1026	0.030*	
C18	0.65246 (17)	0.76107 (11)	0.14473 (10)	0.0232 (4)	
H18	0.7119	0.7859	0.1162	0.028*	
C19	0.60653 (15)	0.66784 (10)	0.23445 (9)	0.0158 (3)	

### Atomic displacement parameters (Å^2^)

**Table:**

	*U*^11^	*U*^22^	*U*^33^	*U*^12^	*U*^13^	*U*^23^
Sn1	0.01411 (7)	0.02073 (8)	0.01692 (8)	−0.00099 (5)	−0.00013 (4)	0.00119 (4)
Cl1	0.0225 (2)	0.0226 (2)	0.0297 (2)	0.00080 (18)	−0.00556 (19)	0.00552 (18)
O1	0.0125 (6)	0.0273 (7)	0.0223 (7)	0.0028 (5)	0.0014 (5)	0.0027 (5)
N1	0.0132 (7)	0.0216 (8)	0.0209 (8)	0.0025 (6)	0.0013 (6)	0.0014 (6)
N2	0.0111 (6)	0.0226 (8)	0.0212 (8)	0.0013 (6)	−0.0007 (6)	0.0016 (6)
C1	0.0192 (9)	0.0256 (9)	0.0224 (9)	−0.0051 (7)	−0.0019 (7)	−0.0006 (7)
C2	0.0197 (9)	0.0216 (9)	0.0223 (10)	−0.0034 (7)	−0.0026 (7)	−0.0057 (7)
C3	0.0206 (8)	0.0270 (10)	0.0215 (10)	0.0010 (8)	−0.0028 (8)	−0.0045 (7)
C4	0.0281 (10)	0.0289 (10)	0.0211 (10)	−0.0017 (8)	−0.0031 (8)	−0.0012 (8)
C5	0.0261 (9)	0.0334 (11)	0.0237 (10)	−0.0050 (9)	0.0040 (8)	−0.0080 (8)
C6	0.0210 (9)	0.0320 (11)	0.0292 (11)	0.0026 (8)	−0.0008 (8)	−0.0098 (8)
C7	0.0235 (9)	0.0232 (9)	0.0247 (10)	0.0001 (8)	−0.0039 (7)	−0.0043 (7)
C8	0.0196 (9)	0.0300 (10)	0.0191 (9)	−0.0004 (8)	0.0027 (7)	0.0010 (8)
C9	0.0224 (9)	0.0274 (10)	0.0150 (9)	0.0004 (8)	0.0039 (7)	−0.0015 (7)
C10	0.0290 (10)	0.0244 (9)	0.0168 (9)	0.0025 (8)	−0.0019 (7)	−0.0013 (7)
C11	0.0231 (9)	0.0374 (12)	0.0208 (10)	0.0008 (8)	−0.0020 (8)	−0.0052 (8)
C12	0.0324 (10)	0.0301 (11)	0.0235 (10)	−0.0094 (9)	0.0042 (8)	−0.0053 (8)
C13	0.0405 (12)	0.0246 (10)	0.0253 (10)	0.0011 (9)	0.0036 (9)	−0.0002 (8)
C14	0.0267 (10)	0.0296 (11)	0.0233 (10)	0.0061 (8)	0.0003 (8)	−0.0016 (8)
C15	0.0131 (8)	0.0219 (9)	0.0186 (9)	0.0011 (7)	0.0000 (7)	−0.0031 (7)
C16	0.0180 (9)	0.0261 (10)	0.0234 (10)	0.0062 (8)	−0.0020 (7)	0.0011 (7)
C17	0.0248 (9)	0.0265 (10)	0.0226 (10)	0.0055 (8)	−0.0006 (8)	0.0059 (8)
C18	0.0241 (9)	0.0233 (9)	0.0223 (10)	0.0014 (8)	0.0031 (8)	0.0050 (7)
C19	0.0139 (7)	0.0185 (8)	0.0151 (9)	0.0014 (6)	−0.0007 (6)	−0.0034 (6)

### Geometric parameters (Å, °)

**Table:**

Sn1—N2	2.0821 (15)	C6—H6	0.9500
Sn1—C8	2.1573 (19)	C7—H7	0.9500
Sn1—C1	2.1604 (18)	C8—C9	1.502 (3)
Sn1—O1	2.2187 (12)	C8—H8A	0.9900
Sn1—Cl1	2.4836 (4)	C8—H8B	0.9900
O1—C15	1.333 (2)	C9—C14	1.392 (3)
N1—C19	1.343 (2)	C9—C10	1.396 (3)
N1—C18	1.370 (2)	C10—C11	1.384 (3)
N1—H1	0.878 (9)	C10—H10	0.9500
N2—C19	1.331 (2)	C11—C12	1.386 (3)
N2—H2	0.871 (9)	C11—H11	0.9500
C1—C2	1.500 (3)	C12—C13	1.383 (3)
C1—H1A	0.9900	C12—H12	0.9500
C1—H1B	0.9900	C13—C14	1.391 (3)
C2—C7	1.396 (3)	C13—H13	0.9500
C2—C3	1.396 (3)	C14—H14	0.9500
C3—C4	1.388 (3)	C15—C16	1.368 (3)
C3—H3	0.9500	C15—C19	1.438 (2)
C4—C5	1.386 (3)	C16—C17	1.417 (3)
C4—H4	0.9500	C16—H16	0.9500
C5—C6	1.386 (3)	C17—C18	1.352 (3)
C5—H5	0.9500	C17—H17	0.9500
C6—C7	1.389 (3)	C18—H18	0.9500
			
N2—Sn1—C8	109.18 (7)	C2—C7—H7	119.6
N2—Sn1—C1	108.13 (7)	C9—C8—Sn1	105.93 (12)
C8—Sn1—C1	141.43 (7)	C9—C8—H8A	110.6
N2—Sn1—O1	75.58 (5)	Sn1—C8—H8A	110.6
C8—Sn1—O1	89.40 (6)	C9—C8—H8B	110.6
C1—Sn1—O1	90.59 (6)	Sn1—C8—H8B	110.6
N2—Sn1—Cl1	88.21 (4)	H8A—C8—H8B	108.7
C8—Sn1—Cl1	95.23 (5)	C14—C9—C10	118.10 (18)
C1—Sn1—Cl1	95.36 (5)	C14—C9—C8	121.45 (17)
O1—Sn1—Cl1	163.77 (3)	C10—C9—C8	120.25 (17)
C15—O1—Sn1	113.14 (10)	C11—C10—C9	120.81 (18)
C19—N1—C18	122.68 (15)	C11—C10—H10	119.6
C19—N1—H1	114.7 (14)	C9—C10—H10	119.6
C18—N1—H1	122.6 (14)	C10—C11—C12	120.59 (19)
C19—N2—Sn1	116.32 (11)	C10—C11—H11	119.7
C19—N2—H2	117.0 (15)	C12—C11—H11	119.7
Sn1—N2—H2	125.7 (15)	C13—C12—C11	119.29 (19)
C2—C1—Sn1	106.34 (12)	C13—C12—H12	120.4
C2—C1—H1A	110.5	C11—C12—H12	120.4
Sn1—C1—H1A	110.5	C12—C13—C14	120.17 (19)
C2—C1—H1B	110.5	C12—C13—H13	119.9
Sn1—C1—H1B	110.5	C14—C13—H13	119.9
H1A—C1—H1B	108.7	C13—C14—C9	121.04 (19)
C7—C2—C3	118.06 (18)	C13—C14—H14	119.5
C7—C2—C1	121.07 (18)	C9—C14—H14	119.5
C3—C2—C1	120.77 (17)	O1—C15—C16	125.95 (16)
C4—C3—C2	121.08 (18)	O1—C15—C19	115.38 (15)
C4—C3—H3	119.5	C16—C15—C19	118.65 (16)
C2—C3—H3	119.5	C15—C16—C17	120.23 (17)
C5—C4—C3	120.27 (19)	C15—C16—H16	119.9
C5—C4—H4	119.9	C17—C16—H16	119.9
C3—C4—H4	119.9	C18—C17—C16	119.72 (18)
C6—C5—C4	119.25 (19)	C18—C17—H17	120.1
C6—C5—H5	120.4	C16—C17—H17	120.1
C4—C5—H5	120.4	C17—C18—N1	119.96 (17)
C5—C6—C7	120.60 (18)	C17—C18—H18	120.0
C5—C6—H6	119.7	N1—C18—H18	120.0
C7—C6—H6	119.7	N2—C19—N1	123.10 (15)
C6—C7—C2	120.73 (19)	N2—C19—C15	118.18 (15)
C6—C7—H7	119.6	N1—C19—C15	118.71 (16)
			
N2—Sn1—O1—C15	9.88 (12)	Sn1—C8—C9—C14	93.23 (18)
C8—Sn1—O1—C15	119.87 (12)	Sn1—C8—C9—C10	−81.55 (18)
C1—Sn1—O1—C15	−98.71 (12)	C14—C9—C10—C11	0.1 (3)
Cl1—Sn1—O1—C15	13.0 (2)	C8—C9—C10—C11	175.01 (17)
C8—Sn1—N2—C19	−94.25 (14)	C9—C10—C11—C12	0.6 (3)
C1—Sn1—N2—C19	75.77 (14)	C10—C11—C12—C13	−0.4 (3)
O1—Sn1—N2—C19	−10.04 (12)	C11—C12—C13—C14	−0.3 (3)
Cl1—Sn1—N2—C19	170.83 (13)	C12—C13—C14—C9	0.9 (3)
N2—Sn1—C1—C2	−1.11 (14)	C10—C9—C14—C13	−0.8 (3)
C8—Sn1—C1—C2	163.69 (12)	C8—C9—C14—C13	−175.65 (18)
O1—Sn1—C1—C2	73.91 (13)	Sn1—O1—C15—C16	170.06 (16)
Cl1—Sn1—C1—C2	−90.98 (12)	Sn1—O1—C15—C19	−8.36 (19)
Sn1—C1—C2—C7	85.78 (19)	O1—C15—C16—C17	−177.14 (17)
Sn1—C1—C2—C3	−90.42 (18)	C19—C15—C16—C17	1.2 (3)
C7—C2—C3—C4	−1.1 (3)	C15—C16—C17—C18	−1.6 (3)
C1—C2—C3—C4	175.23 (18)	C16—C17—C18—N1	0.2 (3)
C2—C3—C4—C5	0.6 (3)	C19—N1—C18—C17	1.7 (3)
C3—C4—C5—C6	0.4 (3)	Sn1—N2—C19—N1	−169.87 (13)
C4—C5—C6—C7	−0.8 (3)	Sn1—N2—C19—C15	9.3 (2)
C5—C6—C7—C2	0.3 (3)	C18—N1—C19—N2	177.05 (17)
C3—C2—C7—C6	0.7 (3)	C18—N1—C19—C15	−2.1 (3)
C1—C2—C7—C6	−175.64 (17)	O1—C15—C19—N2	−0.1 (2)
N2—Sn1—C8—C9	−8.06 (14)	C16—C15—C19—N2	−178.61 (17)
C1—Sn1—C8—C9	−172.75 (12)	O1—C15—C19—N1	179.10 (15)
O1—Sn1—C8—C9	−82.56 (12)	C16—C15—C19—N1	0.6 (3)
Cl1—Sn1—C8—C9	81.86 (12)		

### Hydrogen-bond geometry (Å, °)

**Table:**

*D*—H···*A*	*D*—H	H···*A*	*D*···*A*	*D*—H···*A*
N1—H1···O1^i^	0.88 (1)	1.87 (1)	2.726 (2)	165 (2)

Symmetry codes: (i) *x*+1/2, *y*, −*z*+1/2.
